# Adaptation and validation of the neighbourhood environment walkability scale for German-speaking youth (NEWS-Y-G)

**DOI:** 10.1186/s12889-026-26590-3

**Published:** 2026-02-13

**Authors:** Daniel A. Scheller, Andreas Humpe, Filip Mess, Viviane Nogueira de Zorzi, Cassiano Ricardo Rech, Joachim Bachner

**Affiliations:** 1 TUM School of Medicine and Health, Department Health and Sport Sciences, Associate Professorship of Didactics in Sport and Health, Technical University of Munich, Georg-Brauchle-Ring 62, Munich, 80992 Germany; 2 Munich University of Applied Sciences, Schachenmeierstraße 35, München, 80636 Germany; 3 Postgraduation Program in Physical Education, Federal University of Santa Catarina, Street Eng. Agronômico Andrei Cristian Ferreira, Trindade, Florianópolis, 88040-900 Brazil

**Keywords:** Physical activity, Adolescents, Neighbourhood, Environment, Perception, Walkability, Questionnaire, Reliability, Validity, Urban planning

## Abstract

**Background:**

Adolescents’ physical activity is related to characteristics of their neighbourhood environment. Understanding this relationship can help identify ways to promote this important health-related behaviour. The Neighbourhood Environment Walkability Scale for Youth (NEWS-Y) is the most prominent questionnaire to measure subjective perceptions on the neighbourhood environment that might have an influence on their physical activity. This study describes the development of the NEWS-Y for German-speaking adolescents (called NEWS-Y-G) and the evaluation of its reliability and validity.

**Methods:**

The NEWS-Y-G was adapted and validated through a five-step process. First, the original NEWS-Y was translated into German using a back-translation technique. Participatory methods were then used to establish content validity by identifying environmental aspects specifically relevant to the German context. Third, ten cognitive interviews with adolescents were conducted for ensuring understandability before piloting the questionnaire with 587 adolescents. Of these, 249 participants completed the questionnaire twice, allowing for a test-retest reliability evaluation of subscales and individual items by using intraclass correlation coefficients (ICCs). Finally, construct validity was tested through correlations among subscales and associations with self-reported physical activity levels of participants.

**Results:**

The NEWS-Y-G showed semantic equivalence with the original version. Five items were removed and twelve items were added, resulting in 74 items across the nine subscales: land use mix-diversity, recreational facilities, residential density, land use mix-access, street connectivity, walking/cycling facilities, aesthetics, traffic- and crime safety. The test-retest reliability of subscales (ICC range = 0.81–0.94) and individual items (ICC range = 0.32– 0.94) in the NEWS-Y-G was moderate to almost perfect. Moderate correlations were observed among several NEWS-Y-G subscales. Only two subscales – recreational facilities and aesthetics – showed statistically significant, albeit very weak, associations with self-reported physical activity (both ρ = 0.13, *p* < 0.01), while no significant associations were found for the other seven subscales (ρ = -0.08 to 0.03, *p* > 0.05).

**Conclusion:**

The NEWS-Y-G questionnaire demonstrated good test-retest reliability and provided preliminary evidence of validity. While this study supports its use in measuring subjective walkability among adolescents, further studies in German-speaking regions are needed to confirm its validity in relation to physical activity, accounting for different mobility types as well as gender and socioeconomic disparities. The NEWS-Y-G is a valuable tool for evaluating the neighbourhood environment’s impact on adolescents, providing results that can inform urban planning and research to promote physical activity.

**Supplementary Information:**

The online version contains supplementary material available at 10.1186/s12889-026-26590-3.

## Background

Physical activity (PA) enhances the physical and mental health of children and youth [1]. However, physical inactivity among children and adolescents remains a public health concern with less than 20% of adolescents aged 11 to 17 years worldwide meeting the World Health Organization’s (WHO) PA recommendation of 60 min per day [2]. In Germany, about 17% of boys and 13% of girls aged six to 17 years meet this recommendation, with data before and after the pandemic confirming a consistently low level of PA [3, 4].

In the context of PA promotion in adolescents, evidence-based solutions focus on supportive schools, the social and digital environment as well as multipurpose urban spaces [5]. Consequently, the National Recommendations for Physical Activity and Physical Activity Promotion of Germany highlight the importance of environmental approaches and activity-friendly urban planning [6]. Safe access to (green) spaces, recreational (play) areas and public sports facilities that promote PA including bike- as well as pedestrian-friendly urban environments are measures for urban planners to create PA-supportive environments according to the needs of the young [6].

Environmental neighbourhood characteristics that are related to one’s PA level are often summarised under the term “walkability” [7]. In public health, walkability extends beyond its literal meaning to walk for transport or leisure and serves as a proxy for the activity friendliness of a neighbourhood or city [8]. Many studies indicate that a higher walkability in neighbourhoods is associated with more active lifestyles and healthier weight statuses among young and adult populations [9, 10].

The most commonly used walkability measures in research and urban planning can be both objective and subjective. Objective measures typically involve geographic information systems (GIS) or street audits to quantify physical attributes of the environment [11]. For example, the walkability index is a combined measure of land-use-mix, residential density and street connectivity in a certain area [12]. This index has been positively associated with overall PA levels in adults and the elderly [13]. The availability of sidewalks, determined by using GIS, correlated with daily step counts in the elderly [14]. For younger populations, however, it seems that a broader understanding of walkability is needed. For example, an umbrella review found no significant association between the walkability index and children’s PA levels [13]. This finding suggests that factors besides sidewalks, such as access to leisure areas or subjectively perceived safety, need to be considered [13, 15].

Subjective walkability measures can offer benefits that may outweigh the shortcomings of objective measures. Subjective tools measure individuals’ perceptions of their neighbourhood environment in the form of questionnaires [16]. Factors such as the aesthetics of the landscape and the street security are suggested to be included in those questionnaires, as these might influence young people’s willingness to walk and exercise [9]. Among those subjective measures, the Neighbourhood Environment Walkability Scale for Youth (NEWS-Y) is the most comprehensive tool used internationally to assess perceived walkability and its impact on youth PA [17]. The NEWS-Y questionnaire consists of the nine subscales land use mix-diversity, recreational facilities, residential density, land use mix-access, street connectivity, walking/cycling facilities, aesthetics, traffic safety and crime safety [17]. Several NEWS-Y dimensions – such as residential density, street connectivity and land-use mix-diversity – are comparable to objective walkability measures and could in principle be quantified through GIS. However, other dimensions, including aesthetics, perceived safety, or the presence of social environments, are subjective and cannot be meaningfully evaluated through objective measures alone – which makes the NEWS-Y essential for youth’s perspectives.

Since its development in 2009, the NEWS-Y has been adapted for the use on six continents participating in the International Physical Activity and Environment Network (IPEN) study, using data from 15 countries to examine associations of perceived neighbourhood environment attributes with adolescents’ PA [18]. The involved countries in the IPEN study did not use identical versions of the original English NEWS-Y, which consists of 67 items, but developed a scoring protocol (NEWS-Y-IPEN) to enable between-country comparability. Despite its widespread use in international studies, a comprehensive German version of the NEWS-Y (NEWS-Y-German) is missing, except for a shortened scale with eight items [19]. The lack of a validated German version limits the ability to conduct comparable research on neighbourhood perceptions in German-speaking countries. This impedes the development of targeted interventions to improve perceived walkability and related PA levels among German youth [20]. Adapting the NEWS-Y requires an involvement of the of the target group, as has been illustrated in the development of a Chinese NEWS for children (NEWS-CC), whose validity and reliability were confirmed via confirmatory factor analysis (CFA) and intraclass correlation coefficients (ICCs) following a participatory adaptation phase of the questionnaire.

The aim of this study is to develop the NEWS-Y-German (NEWS-Y-G) through a similarly thorough procedure, thus ensuring it is a reliable and valid tool for evaluating perceived walkability in German-speaking youth. By doing so, this research will provide a valuable tool for evaluating subjective walkability in the German-speaking region and enable national as well as international comparisons. The further application of the NEWS-Y-G in future studies will provide practical implications for urban planners to promote environments that support active lifestyles among adolescents in Germany.

## Methods

### Original version of the NEWS-Y

The original English version of the NEWS-Y consists of 67 items structured in nine subscales that measure different aspects of the neighbourhood environment [17]. The subscales land use mix-access, street connectivity, walking/cycling facilities, aesthetics, traffic safety and crime safety use a 4-point Likert scale (1 = strongly disagree; 4 = strongly agree). Land use mix-diversity and recreational facilities are checklists that assess the proximity of neighbourhood destinations and amenities, with five walking distance options ranging from 1 to 5 min to over 30 min, including an “I don’t know” option. Residential density is assessed by four items asking about different housing types in the neighbourhood using a 5-point scale (1 = there are none XX; 5 = all residences are XX; with XX being, for example, stand-alone family houses or apartments). There are weights applied to each type of housing to estimate the residential density, e.g. a weight with the factor of “2” for duplex homes with typically two parties living in such houses.

The development of the NEWS-Y-G was conducted in the following five steps to ensure its reliability and validity with the help of the target group. The initial three steps and the final step focused on ensuring validity by (1) achieving semantic equivalence between the original and the German NEWS-Y version through a back-translation technique; (2) identifying environmental aspects specifically relevant to the German context that might not be covered in the original NEWS-Y using participatory methods with youth; (3) ensuring that adolescents fully understand the content of the questionnaire by using cognitive interviews. For testing reliability, (4) a test-retest design was used with a two-week period for adolescents to fill out the questionnaire twice. As a final step, (5) the intercorrelations between the subscales and their associations with self-reported PA were examined. In the following, the methodological procedure according to these five steps is described in more detail.

### Step 1: translation of the original NEWS-Y

A translation and adaptation of the NEWS-Y required experts proficient in English and the target language (here German) as well as the participation of the target group and pre-testing the developed NEWS tool with them. For instance, in the development of the NEWS-CC, a back-translation technique was used following interviews with children from Hong Kong by using a nominal group technique for adapting the NEWS-Y questionnaire [21]. The translation of the NEWS-Y from English to German to ensure semantic equivalence adhered to the process recommended by the WHO, which also had been used in other NEWS translations [21, 22]. Initially, two bilingual individuals, one with a background in translation and the other in health sciences, independently translated the original English version of the NEWS-Y into German (forward translation). The two translations were then combined with the forward-translators to produce one synthesised German version. This synthesised translation was subsequently back-translated into English by two other bilingual speakers with expertise in health and sport sciences (back translation). The back-translated versions were then synthesised with the back-translators. The synthesised back-translated version was then tested for semantic equivalence with the original English questionnaire using Flaherty’s 3-Point Scale to ensure the meaning of each item remained consistent [23]. DAS scored each item from “1” to “3”, where “3” indicated identical meaning, “2” indicated similar meaning and “1” indicated different meanings. Items that were not rated with “3” were revised by JB and DAS to decide on a final formulation of the items.

### Step 2: adaptations to the NEWS-Y according to the German context

The adaptations to the NEWS-Y to ensure regional relevance, e.g. with regard to local environmental attributes, were guided by the use of participatory methods, which involve engaging the target population. These methods were implemented in the WALKI-MUC project, which, amongst other research aims, evaluated subjective walkability perceived by children and adolescents living in urban environments of Munich, Germany [24]. To identify aspects of the natural and built environment that are relevant for the activity friendliness of a neighbourhood (and thus should be considered in the NEWS-Y-G), 93 children and adolescents aged six to 17 years from six neighbourhoods of Munich with different socio-economic statuses (SES) and levels of objectively-measured walkability were engaged in walking interviews. During these interviews, participants discussed their neighbourhoods, focusing on places that encouraged or discouraged their PA. Their input was enhanced with photovoice techniques and mapping exercises during focus group discussions. The semi-structured interview protocol that was used in the walking interviews as well as the results of the photovoice study are documented elsewhere [24, 25]. Walking interview statements in which the participants referred to natural, built or other environmental aspects that they deemed relevant for how they perceive the walkability of their urban living environment were extracted and aggregated. These environmental aspects were sorted by frequency of mention across all participants. An aspect was included as a new item in the NEWS-Y-G if it surpassed the threshold of five mentions by different participants. Additionally, also aspects that according to the participants’ statements clearly represented everyday activities related to the natural or built environment were addressed by adding new items. On the other hand, similarly to the development of the NEWS-IPEN [18], items that were not deemed highly relevant to adolescents in the German context were omitted to reduce the length of the questionnaire (e.g. YMCA).

### Step 3: cognitive interviews

Cognitive interviews were conducted with ten participants aged seven to 18 years (five male and five female participants with a mean age of 14.3 years, SD = 3.2) to evaluate the newly developed questionnaire for understandability. Participants were recruited through the networks involved in the WALKI-MUC project, which also played a role in adapting the NEWS-Y. Prior to the interviews, all participants independently completed the NEWS-Y-G questionnaire and were instructed to mark any items they found difficult to understand or inappropriate. Subsequently, cognitive interviews were conducted by DAS and NM individually with each participant using a structured interview guide specifically designed for this purpose. In this guide, items had been pre-selected for discussion that had already been suspected of causing difficulties. Yet, participants also had the opportunity to point out incomprehensible items themselves. Participants were given the chance to help re-wording the marked items and their responses were documented.

### Steps 4 and 5: test-retest of the NEWS-Y-G and examination of its construct validity

The newly developed NEWS-Y-G was pilot-tested with a convenience sample of adolescent participants in Germany. For this purpose, undergraduate students majoring in tourism at Munich University of Applied Sciences were tasked with recruiting German-speaking school-aged adolescents. Each student was provided with printed versions of the NEWS-Y-G questionnaire to pass on to potential participants.

To assess test-retest reliability, participants were asked to complete the questionnaire twice without assistance, with a two-week interval between the first and second completion. After the first completion, the questionnaires were collected to avoid influencing the participants’ responses during the second completion. Besides the NEWS-Y-G items, data collected included gender, age, postal code, school type, self-reported PA, duration of residence (how many years a participant had already lived in the respective neighbourhood) and the body mass index (BMI).

Physical activity reporting followed WHO recommendations [26], as well as for the BMI [27]. Physical activity was measured using a single self-report item inquiring on how many days per week individuals engage in at least 60 min of moderate-to-vigorous PA. Participants were informed that this referred to any PA, including organised sports, active outdoor play or active transport such as cycling. Single-item PA measures of this type are commonly applied in international youth studies and have demonstrated acceptable reliability and validity for children and adolescents, particularly for ranking individuals according to activity level in epidemiological research [28]. Multi-item PA instruments are considerably longer and would have increased respondent burden in an already extensive questionnaire. Given the young age of the participants, it was aimed to keep the survey as simple and accessible as possible.

To determine the test-retest reliability of the items, a two-way-mixed effects model for intraclass correlation coefficients (ICCs) was calculated. Many of the previous NEWS-Y adaptation studies that reported their ICC procedures applied the one-way model [21, 29–31]. However, according to the present study design and aims, current guidelines for ICC selection and reporting recommend the two-way-mixed effects model [32]. As the same participants completed the questionnaire twice, with measurement occasions treated as fixed and individuals as random, a two-way-mixed effects model with absolute agreement was appropriate. The absolute agreement definition allowed to evaluate whether subscale scores remained stable in their exact values between test and retest. For this purpose, scores were calculated for each of the subscales based on the mean values of all items within a subscale – except for the score of subscale C, which was calculated based on the formula: “C1 + C2*12 + C3*2 + C4*25” [17]. The interpretation of ICC values for reliability was: 0.00–0.20 as slight, 0.21–0.40 as fair, 0.41–0.60 as moderate, 0.61–0.80 as substantial and 0.81–1.00 as almost perfect reliability [33]. Items with an ICC value of 0.40 or less were considered to have poor reliability and require reconsideration.

Tests of internal consistency using Cronbach’s alpha and CFA like in other validation studies (e.g. the one of the NEWS-CC) were not performed for the NEWS-Y-G. The NEWS-Y-G scale is primarily intended to measure environmental characteristics and as such it is not a psychometric scale in the traditional sense. In this context, Reimers et al. (2013) emphasise that for formative constructs, such as the living environment, statistical methods such as calculating Cronbach’s alpha or conducting a factorial analysis are not useful, in contrast to their fit for reflective psychological constructs [34]. A low alpha value would not necessarily indicate a poor quality of the scale, but merely reflect the diversity of the characteristics measured in a certain subscale.

Validity was evaluated following the COSMIN framework for measurement properties, which distinguishes between content-related and construct-related aspects of validity [35]. Face validity, as part of content validity, was assured through the qualitative approaches applied in Steps 2 and 3. In this regard, the used participatory methods and cognitive interviews with adolescents should ensure the relevance and comprehensibility of items for the target group. Construct validity was examined using a hypothesis-testing approach, as recommended by COSMIN. Specifically, it was hypothesised a priori that (a) there would be intercorrelations between subscales, reflecting the multidimensional nature of walkability and (b) subscales would show positive associations with self-reported PA, as suggested by literature. Construct validity was therefore evaluated through inter-subscale correlations and correlations between subscales and self-reported PA, both examined using Spearman correlations. These analyses were not adjusted for potential confounders such as age, gender, or socioeconomic status.

For all tests, IBM SPSS statistics (version 29.0) was used and a p-value of 0.05 was considered for statistical significance in some analyses.

### Ethical considerations

All participants that were involved in one or more of the steps above received project information and consent forms via educational actors, students, or directly by mail or e-mail. For participation, one parent/legal guardian and the participant had to sign a declaration of consent. Written consent was obtained and documented from all participants for their inclusion in the study. The ethics committee of the Technical University of Munich has approved the study protocol (reference number 77/22 S).

## Results

### Semantic equivalence and adaptations to the NEWS-Y

On Flaherty’s 3-Point Scale, no item received a score of “1” in the evaluation of semantic equivalence, which indicates that there were no differences in meaning between the items of the NEWS-Y-G and the original English questionnaire. Eight items were rated with a score of “2”, suggesting similar meanings. The majority of items (59 items) were rated with a score of “3”, confirming that their meanings were identical across both versions.

The adaptation process helped identify necessary adjustments for the German context, resulting mainly in modifications to the subscales A “Land use mix-diversity” and B “Recreational facilities”, but also some changes to other subscales. The adaptations are summarised in Table 1.

**Table 1:** Adapted subscales of the NEWS-Y-G with items removed from and added to the original NEWS-Y

Subscale	Removed items	Added items
A. Land use mix-diversity	Video store	Shopping mall
		Kindergarten
B. Recreational facilities	Basketball court	Soccer court
	YMCA	Public park
	Small public park	Indoor gym
	Large public park	Calisthenics park / parkour site
		Climbing/bouldering facility
		Bench
		Mountain/hill
F. Walking facilities		Sidewalks separated from bike lanes
G. Aesthetics		Litter
H. Traffic safety		Shade

The walking interviews and the cognitive interviews highlighted inconsistent perceptions and varying experiences with several aspects addressed in the original NEWS-Y (e.g. hardware store) and the German adaptations (e.g. public market in addition to vegetable store) in the subscale land use mix-diversity. The item “Video store” was removed and some items have been reworded to make them easier to understand where this was considered necessary. Furthermore, the subscale was updated with two new items: “Mall” and “Kindergarten”. The “Coffee place” was expanded to include “Ice cream parlour”.

The subscale recreational facilities underwent significant changes. “YMCA” was removed, “Soccer court” was added as a standalone item, whereas “Basketball court” was grouped with other sports facilities, contrary to the original American NEWS. “Climbing facilities” was added and “Parkour” was included as a separate item. The new items “Calisthenics Park/Fitness Park” and “Fitness Studio/Gym” were added for outdoor and indoor sports, respectively. “Small public park” and “Large public park” were merged into “Public park” because a distinction was difficult for the adolescents in the cognitive interviews. Sitting facilities in form of “Benches” and “Mountain/hill” were also included.

The residential density subscale required minor adjustments in wording for clarity according to German housing. However, the weights applied to each type of housing to calculate residential density in the neighbourhood were left unchanged to the original formula of the NEWS-Y.

Land use mix-access and street connectivity subscales received no changes. In the subscale walking/cycling facilities, a new item on “Bike lanes separated from sidewalks” was added.

A new item was added to the aesthetics subscale that addressed the presence of “Litter”. Items on “Interesting things” and “Beautiful natural things” in the neighbourhood were clarified with examples mentioned in the interviews. The traffic safety subscale was given a new item on the availability of “Shade”.

The item in the crime safety subscale originally assessing a “High crime rate” was reworded to “Committed criminal offenses” and relocated to be asked after the items addressing feelings of safety in the neighbourhood to reduce priming effects in this subscale.

Overall, five original items were removed from and twelve new items were added to the NEWS-Y, resulting in 74 items structured in nine subscales in the NEWS-Y-G. Higher scores in all subscales indicate a higher perceived walkability. The time taken for questionnaire completion was recorded before the cognitive interviews, averaging in 21 min, with the shortest completion time at 14 min and the longest at 30 min.

### Sample characteristics

The overall test sample who provided questionnaire data included 587 participants (see Table 2). Of these participants, 321 (54.7%) were female, 258 (44.0%) were male and eight (1.4%) identified as neither of them. The mean age of the sample was 14.9 years (SD = 1.6). Participants attended all different types of German secondary schools, which represent low (Mittelschule), medium (Realschule), high (Gymnasium) and other levels of formal education. In total, 558 participants provided their postal codes, with 304 adolescents indicating their residence in Munich’s urban area, while 254 participants lived in the metropolitan region around Munich or in other cities across Germany. The average duration of residence was 11.2 years (SD = 5.0).

**Table 2:** Characteristics of study sample

Variables	N	%(CI_95%_)
Gender (n=587)		
Female	321	54.7 (50.6-58.7)
Male	258	44.0 (40.0-48.0)
Diverse	8	1.4 (0.7-2.7)
School Type (n=514)		
Mittelschule	110	21.4 (18.1-25.2)
Realschule	146	28.4 (24.7-32.5)
Gymnasium	219	42.6 (38.4-46.9)
Others	39	7.6 (5.7-10.2)
Body Mass Index^a^(n=559)		
Underweight	83	14.9 (12.1-18.1)
Normalweight	392	70.1 (66.2-73.8)
Overweight	67	12.0 (9.5-15.0)
Obesity	17	3.0 (1.9-4.8)
Physical Activity levels^b^(n=584)		
Insufficiently active	512	87.7 (84.7-90.1)
Active	72	12.3 (9.9-15.3)
	**Mean**	**SD**
Age in years (n=563)	14.9	1.6
Weight in kg (n=559)	60.3	13.1
Height in cm (n=582)	168.7	10.5
Residential Time in years (n=564)	11.2	5.0

*SD * Standard deviation

^a^BMI Cutoff points (according to WHO 2007 in percentiles) (<15: underweight;  ≥ 15 to < 85:  normalweight;  ≥ 85 to < 97: overweight); ≥ 97: obesity)

^b^Insufficiently active  (less than 7 days with 60 minutes of moderate-to-vigorous PA per day in the last week), Active (7 days with 60 minutes/daily)

All NEWS-Y-G subscales showed adequate variability and covered almost the full range of the possible scale scores at baseline (Table 3).

**Table 3:** Descriptive statistics of NEWS-Y-G subscale scores at baseline

Subscale [score range]	*N*	Min	Max	Mean (SD)
A. Land use mix-diversity [1–6]	586	1.0	6.0	4.1 (0.8)
B. Recreational facilities [1–6]	585	1.8	6.0	4.0 (0.8)
C. Residential density [0-200]	579	40.0	199.0	109.6 (34.8)
D. Land use mix-access [1–4]	559	1.2	4.0	3.2 (0.5)
E. Street connectivity [1–4]	567	1.0	4.0	2.8 (0.6)
F. Walking/cycling facilities [1–4]	571	1.0	4.0	2.5 (0.6)
G. Aesthetics [1–4]	574	1.0	4.0	2.4 (0.5)
H. Traffic safety [1–4]	551	1.5	3.88	2.9 (0.4)
I. Crime safety [1–4]	571	1.0	4.0	3.4 (0.6)
NEWS-Y-G score^a^	505	6.8	25.2	15.0 (4.0)

Baseline refers to the first administration of the NEWS-Y-G questionnaire. Subscales differ in their possible score ranges due to varying numbers of items and response formats

^a^Mean value of all subscales

The sample that provided questionnaire data twice, at both test and retest, comprised 249 adolescents, including 114 boys and 135 girls aged between seven and 18 years. The mean age was 14.9 years (SD = 1.6), which was consistent with the sample that completed the NEWS-Y-G only once.

### Test-retest intraclass correlation coefficients (ICCs)

Reliability analysis was conducted for each subscale and item of the NEWS-Y-G, as shown in Table 4.

**Table 4:** Test-retest ICCs^a^ of subscales^b^ and individual items of the NEWS-Y-G

Subscale / Item	ICC (95%-CI)
**A. Land use mix-diversity**	**0.94 (0.92–0.95)** ^b^
1. Convenience store	0.70 (0.62–0.77)
2. Supermarket	0.38 (0.21–0.52)
3. Hardware store	0.50 (0.36–0.61)
4. Vegetable store / public market	0.38 (0.20–0.52)
5. Laundry	0.32 (0.13–0.47)
6. Clothing store	0.50 (0.35–0.61)
7. Post office	0.88 (0.85–0.91)
8. Library	0.89 (0.85–0.91)
9. Kindergarten	0.86 (0.83–0.89)
10. Primary school	0.86 (0.81–0.89)
11. Secondary school	0.86 (0.82–0.89)
12. Bookstore	0.87 (0.83–0.90)
13. Fast food restaurant	0.89 (0.85–0.91)
14. Coffee shop / ice parlor	0.87 (0.83–0.90)
15. Bank	0.92 (0.89–0.93)
16. Restaurant	0.82 (0.77–0.86)
17. Shopping mall	0.89 (0.86–0.92)
18. Pharmacy	0.86 (0.81–0.89)
19. Hair salon	0.91 (0.88–0.93)
20. Any office/construction site	0.87 (0.83–0.90)
21. Public transit station	0.70 (0.62–0.77)
**B. Recreational facilities**	**0.92 (0.90–0.94)** ^b^
1. Indoor leisure or sport facility	0.82 (0.77–0.86)
2. Indoor gym	0.88 (0.84–0.91)
3. Calisthenics park / parkour site	0.88 (0.85–0.91)
4. Beach / lake / river / creek	0.89 (0.86–0.91)
5. Hiking / bike trail	0.80 (0.75–0.85)
6. Soccer field	0.88 (0.85–0.91)
7. Other sports field	0.81 (0.75–0.85)
8. Climbing / bouldering facility	0.83 (0.78–0.87)
9. Youth centre	0.92 (0.89–0.94)
10. Swimming pool	0.88 (0.85–0.91)
11. Running track	0.81 (0.76–0.85)
12. School with public sports field	0.80 (0.74–0.84)
13. Public park	0.88 (0.85–0.91)
14. Public playground	0.78 (0.72–0.83)
15. Open place (grass, sands, dirt)	0.83 (0.78–0.87)
16. Bench	0.80 (0.74–0.84)
17. Mountain / hill	0.91 (0.88–0.93)
**C. Residential density**	**0.91 (0.88–0.93)** ^**b**^
1. Detached single-family houses	0.94 (0.93–0.96)
2. Townhouses	0.83 (0.78–0.86)
3. Multi-family / semi-detached houses	0.90 (0.88–0.92)
4. Apartment blocks / high-rise buildings	0.93 (0.90–0.94)
**D. Land use mix-access**	**0.91 (0.88–0.93)** ^b^
1. Easy to walk to shops	0.89 (0.86–0.91)
2. Hard to park	0.86 (0.83–0.90)
3. Easy to go to various places	0.86 (0.82–0.89)
4. Easy to go to public transit station	0.76 (0.70–0.82)
5. Steep streets	0.87 (0.83–0.90)
6. Walking barriers	0.73 (0.65–0.79)
**E. Street connectivity**	**0.81 (0.75–0.85)** ^b^
1. Not many cul-de-sacs	0.77 (0.71–0.82)
2. Short distance between street crossings	0.78 (0.71–0.83)
3. Many alternative paths	0.82 (0.77–0.86)
**F. Walking facilities**	**0.87 (0.84–0.90)** ^b^
1. Sidewalks on most streets	0.83 (0.78–0.86)
2. Sidewalks separated by parked cars	0.73 (0.65–0.79)
3. Sidewalks separated by green space	0.80 (0.75–0.85)
4. Sidewalks separated from bike lanes	0.85 (0.81–0.89)
**G. Aesthetics**	**0.86 (0.81–0.89)** ^b^
1. Trees along streets	0.83 (0.79–0.87)
2. Interesting things	0.81 (0.76–0.85)
3. Beautiful natural things	0.79 (0.72–0.83)
4. Beautiful buildings	0.86 (0.82–0.89)
5. Litter	0.81 (0.76–0.85)
**H. Traffic safety**	**0.87 (0.83–0.90)** ^b^
1. Amount of traffic	0.74 (0.66–0.80)
2. Speed limits	0.80 (0.75–0.85)
3. Speeding	0.84 (0.79–0.87)
4. Good lighting at night	0.84 (0.79–0.87)
5. Easy to see pedestrians or cyclists	0.73 (0.65–0.79)
6. Crosswalks or signals	0.80 (0.74–0.84)
7. Exhaust fumes	0.77 (0.71–0.83)
8. Shades	0.78 (0.71–0.83)
**I. Crime safety**	**0.84 (0.80–0.88)** ^b^
1. Afraid of being outside alone near home	0.82 (0.77–0.86)
2. Afraid of being outside with friends near home	0.67 (0.58–0.75)
3. Afraid of being outside on nearby streets	0.75 (0.68–0.81)
4. Afraid of being outside in nearby parks	0.79 (0.73–0.83)
5. Criminal offenses	0.83 (0.79–0.87)
6. Unsafe to be outdoors at night	0.79 (0.74–0.84)

^a^ICCs calculated by a two-way-mixed effects model

^b^ICCs calculated based on subscale level highlighted in bold

The test-retest reliability of the subscales can be classified as almost perfect (ICC range = 0.81–0.94). Subscale A (Land use mix-diversity) showed a very good overall reliability, however, individual items varied. For instance, items like “Supermarket”, “Vegetable store / Public market” and “Laundry” exhibited only fair reliability. Similarly, the items “Hardware store” and “Clothing store” showed moderate reliability, indicating relatively consistent responses over time but with some variability. The new items “Shopping mall” and “Kindergarten” demonstrated high reliability.

The newly added items in subscale B (Recreational facilities), namely “Soccer courts”, “Public park”, “Indoor gym”, “Calisthenics park / parkour site”, “Climbing/bouldering facility”, “Bench” and “Mountain/hill”, exhibited substantial to almost perfect test-retest reliability, as did the whole subscale.

Subscales C to I also showed almost perfect reliability, including the new items added to subscale F “Sidewalks separated from bike lanes”, subscale G “Litter” and “Shades” in subscale H.

### Testing intercorrelations of the NEWS-Y-G subscales

All subscales were analysed for their interrelations. The values in Table 5 highlight the weak to moderate correlations that were observed. “Land use mix-diversity” (subscale A) showed a moderate positive correlation with subscale B “Recreational facilities” and with subscale D “Land use mix-access”. Subscale D showed also moderate correlations with subscales C “Residential density”, subscale E “Street connectivity” and subscale F “Walking facilities”. Subscale F showed weak correlations with all other subscales, except subscale B, and slightly stronger with subscale C. Notably, subscale C was also moderately associated with subscale A in a positive manner. Subscales H “Traffic safety” and I “Crime safety” also showed a moderate positive correlation.

**Table 5:** Correlations between subscales of the NEWS-Y-G

	A	B	C	D	E	F	G	H	I
A	1								
B	.50*	1							
C	.32*	.11*	1						
D	.42*	.10*	.36*	1					
E	.20*	.10*	.14*	.31*	1				
F	.29*	.075	.36*	.39*	.20*	1			
G	-.03	.18*	-.02	-.01	.05	.20*	1		
H	.08	.03	.01	.22*	.25*	.13*	.04	1	
I	-.09	-.03	-.19*	-.01	.02	-.07	-.09*	.37*	1

Values with a Spearman's ρ greater than 0.3 are highlighted in bold

*p-value < 0.05 (two-tailed)

### Associations of subscales with duration of residence and self-reported physical activity

Weak correlations were observed between the duration of residence and several subscales including recreational facilities (ρ = 0.10, *p* < 0.05), land use mix-access (ρ = -0.11, *p* < 0.05), walking facilities (ρ = -0.18, *p* < 0.01) and aesthetics (ρ = -0.08, *p* < 0.05), indicating that longer residence in an area is mainly associated with lower perceived walkability in these subscales (except for subscale B). Physical activity showed only two statistically significant but small associations with the recreational facilities and the aesthetics subscales (both ρ = 0.13, *p* < 0.01), while all other associations with subscales were non-significant: land use mix-diversity (ρ = -0.02, *p* > 0.05), residential density (ρ = -0.08, *p* > 0.05), land use mix-access (ρ = -0.08, *p* > 0.05), street connectivity (ρ = 0.03, *p* > 0.05), walking facilities (ρ = -0.05, *p* > 0.05), traffic safety (ρ = 0.03, *p* > 0.05) and crime safety (ρ = 0.02, *p* > 0.05).

## Discussion

In this study, the NEWS-Y questionnaire that is used for measuring neighbourhood perceptions regarding walkability was adapted for German-speaking adolescents. The adaptation and validation methodology followed the framework of a similar study, including the use of back-translation of the NEWS-Y, qualitative research methods for tailoring the questionnaire to the target group and a test-retest design [21]. The high level of semantic equivalence of the NEWS-Y-G with the original version confirmed the accuracy of the back-translation process. The results of photovoice and walking interviews with adolescents in their neighbourhoods were used to identify specific items relevant to the German context that were added to the NEWS-Y-G. Moreover, cognitive interviews were conducted with adolescents to verify that the items were interpreted as intended. After adaptation, the questionnaire consisted of 74 items, while maintaining the structure of the original nine subscales of the NEWS-Y. The baseline NEWS-Y-G subscale scores showed adequate variability and no floor or ceiling effects. This indicated that the data were well distributed and suitable for evaluating the questionnaire’s validity and reliability.

### Validity and reliability of the NEWS-Y-G

The reliability and validity of the newly developed NEWS-Y-G were evaluated in a sample of German youth with a sample size that was similar to the original NEWS-Y study and other adaptations of the tool that have followed [17, 18]. The test-retest reliability of the nine subscales of the final NEWS-Y-G can be considered as almost perfect (ICC range = 0.81–0.94), which is comparable to other studies that also investigated the test-retest reliability of the NEWS or similar questionnaires [17, 21, 29–31, 36–38].

On item level, ICC ranged from 0.32 to 0.94 with a relatively low ICC between 0.30 and 0.50 found for the three items “Supermarket”, “Vegetable store / public market” and “Laundry”. Based on their low ICCs, it was decided to include one new item as well as to modify and omit two of these items in the final questionnaire (Additional file 1), respectively. Supermarkets were often confused with drugstores in the walking interviews, which may have led to different stores (thus different walking distances) being reported at the two measurement times causing a low ICC. Therefore, an additional item for “Drugstores” was included. The item “Vegetable store / public market” was reduced to only “Vegetable store” like in the original NEWS-Y, as the cognitive interviews revealed difficulties with the interpretations of public markets. The item “Laundry” was omitted due to its irrelevance in the daily lives of adolescents, as they may not frequently visit these amenities (as indicated in the walking interviews), leading to less consistent perceptions and reporting over time.

The practical use of the NEWS-Y-G lies in measuring environmental characteristics, their relationships to PA and their relevance to the design of PA-friendly neighbourhoods. The validity evaluation of the scale therefore focused on content-related and construct-related aspects, following the COSMIN framework for measurement properties [35]. Content validity (i.e. face validity) was ensured through the qualitative development steps, including participatory methods and cognitive interviews with adolescents, which confirmed the relevance, clarity and comprehensibility of items for the target group. Construct validity was subsequently examined using a hypothesis-testing approach. Based on a priori assumptions, intercorrelations among subscales and associations with self-reported PA were analysed to evaluate whether observed relationships were consistent with theoretical expectations regarding the multidimensional construct of perceived walkability.

The intercorrelations among NEWS-Y-G subscales were weak to moderate, which is theoretically plausible given that each of the nine subscales captures a distinct, though related, environmental attribute of the construct “walkability”. In this context, very high correlations would suggest redundancy between subscales, whereas very low correlations might indicate conceptual fragmentation. Thus, the observed correlations represent preliminary, theory-consistent indications (rather than definitive evidence) of construct validity of the NEWS-Y-G.

Regarding the intercorrelations of subscales, the positive associations of subscale A with B (ρ = 0.50), C (ρ = 0.32) and D (ρ = 0.42) suggest that areas with a more diverse mix of land use also tend to have more recreational facilities in walking proximity, a higher residential density and better access to services, all of which are typically met by historical urban centres. City centres are characterised by a complex synergy of three walkability measures, the so-called urban DMA (Density, Mix and Access): density means more people and places within walking distance; functional land-use-mix encompasses a wider range of walkable destinations, and access includes the availability of transport and traffic between them [39]. Further, the relationships between the subscales of “Residential Density”, “Walking Facilities” (ρ = 0.36) and “Land use mix-access” (ρ = 0.36), the latter being also related to “Street Connectivity” (ρ = 0.31) confirms the principle of urban DMA, with more amenities and services in walkable distance, well-connected streets and safe walking infrastructure facilitating easier access for residents. The correlation of density and amenities is a typical result of the urban planning concept for creating “compact cities”, which encourage sustainable transportation modes such as walking, as highlighted in the United Nations’ Sustainable Development Goals [40, 41]. Additionally, the correlation of the two safety-related subscales H and I (ρ = 0.37) shows that environments perceived as safe for traffic are also perceived as having lower risk for crimes. However, with regard to PA levels, adolescents’ neighbourhood perceptions on safety measured by the NEWS were neither associated in this study, nor in previous studies [42].

Instead, the positive associations between self-reported PA levels and the subscales B “Recreational facilities” (ρ = 0.13) and G “Aesthetics” (ρ = 0.13) reinforce that adolescents’ perceptions on recreational facilities and aesthetic qualities in a neighbourhood are significantly linked to their PA levels, albeit weakly. These findings are in line with the literature introduced earlier and represent an additional, theory-based criterion for validity [13, 15]. However, the small effect sizes provide only limited and preliminary support for the construct validity of the NEWS-Y-G, which requires further evaluation. Longitudinal study designs, ideally combined with analytical approaches that control for potential confounders, would be needed to determine whether and how particular walkability dimensions (measured by one subscale) are causally linked to youth’s PA levels.

In this regard, it can also be assumed that the longer people lived in their neighbourhood, the better they understand and know the area, leading to more accurate perceptions of their surroundings and evaluations of its walkability. Indeed, a longer residence was associated with higher scores in subscale B (ρ = 0.10) indicating greater familiarity with recreational facilities the longer an adolescent has lived in the neighbourhood. Interestingly, a longer residence in a neighbourhood was associated with lower perceived walkability in several subscales (D, F, G), even if the correlations were weak (all below ρ = 0.20). A longitudinal study with adults has already found a lower perceived walkability being associated with longer residence in a neighbourhood [43]. This relationship would need to be further researched in adolescents to determine whether these negative associations are due to an increasing familiarity or potential dissatisfaction with their environment.

### Strengths and limitations

The NEWS-Y-G was developed in five consecutive steps to ensure its reliability and validity by including the target group of adolescents. Content validity was ensured by achieving semantic equivalence between the original and the German NEWS-Y version through back-translation and using participatory methods, including photovoice and walking interviews, to identify German-specific environmental aspects to be included in the questionnaire. Conducting cognitive interviews for clarity was an additional step that was rarely found in other NEWS adaptations. The overall good reliability was confirmed through a test-retest design with a two-week interval. Finally, the relationships between the subscales and their associations with self-reported PA offer preliminary support for the questionnaire’s construct validity, while indicating the need for further confirmation in future studies.

In future evaluations, it should also be checked how the perceived walkability assessed by the NEWS-Y-G correlates with specific PA behaviours, particularly distinguishing between recreational and transport-related PA and different modes of transport such as walking and cycling. Further, participants’ perceptions of their neighbourhood environments should be compared to objectively measured environmental attributes, e.g. with GIS data on walkability.

It is further important to consider gender disparities and SES as factors that may have an influence on perceptions and the relationships with specific activities like leisure walking or transport-related walking. Adolescents’ gender and their SES have shown different associations with environmental factors when objectively measured with GIS and subjectively measured with the NEWS-Y [44]. Therefore, these variables should be included in evaluations with the NEWS-Y-G of larger (representative) samples to confirm its validity across different gender and SES.

The inclusion of parental perspectives could also be beneficial. In this study, a correlation was found between meeting PA guidelines and neighbourhood aesthetics, whereas in the original NEWS-Y study by Rosenberg et al. (2009), this was the case only when parents completed the questionnaire for their children [17]. No such correlation was observed when adolescents filled out the questionnaire themselves. Parents’ neighbourhood perceptions are well-documented to be associated with their children’s overall activity, transport-related PA and play, with this effect being more pronounced for children than adolescents [45–47]. Due to these prior findings, younger children and parents were excluded from the validation process of the NEWS-Y-G. This tool is specifically designed to explore adolescents’ perceptions of the environment to ensure that results are directly relevant to this age group, providing a clearer understanding of how their perceptions influence their PA behaviours. However, parental perceptions of traffic and crime-related safety seem to play a role in the mobility and PA levels of their children [42, 48] and could therefore be considered in future evaluations with the NEWS-Y-G.

### Practical implications for urban planners and practitioners

The NEWS-Y-G emphasises the need for an understanding of walkability that goes beyond traditional objective measures in the context of urban planning. Geographic information system tools and data from so-called secondary sources (e.g. Google Earth or Google Street View) have become the preferred choice for urban planners, as the data is often available and provides a cost-effective alternative [11]. While objective walkability assessments quantify physical attributes such as land-use mix, residential density and street connectivity, subjective measures like the NEWS-Y-G additionally capture individual perceptions on the environment. For this reason, subjective assessments can play a complementary role to objective measures. They reflect how young people perceive their neighbourhood environment, which may be more directly related to behavioural outcomes such as PA. In this context, it is a further validity criterion that the aesthetics subscale of the NEWS-Y-G – a solely subjective dimension – showed a significant association with PA in this study. Although this association was small, it reflects the added value of perceived walkability measures. At the same time, subjective measures are susceptible to individual biases and contextual influences. Objective measures provide standardised and reproducible measures of the built environment instead. Thus, a comprehensive evaluation of walkability should integrate both, subjective and objective measures, to better reflect the living environments of young populations. Here, the NEWS-Y-G provides an opportunity for urban planners to not only evaluate adolescents’ needs regarding their environmental conditions, but also to engage them in the process of creating active neighbourhoods.

## Conclusion

The newly developed NEWS-Y-G tool for measuring subjective walkability showed good test-retest reliability and preliminary validity in a sample of German adolescents. This study describes the individual steps taken to develop the questionnaire and supports its use in the evaluation of neighbourhood environments with adolescents in the German-speaking region. While the instrument effectively measured relevant environmental characteristics, future research should aim to further confirm its validity, taking into account different types of PA, gender differences and SES to ensure its broad applicability. The NEWS-Y-G is a valuable tool for assessing the neighbourhood environment’s impact on adolescents, providing results that can inform urban planning and research aimed at promoting PA among adolescents.

## Supplementary Information

Supplementary Material 1.

Supplementary Material 2.

Supplementary Material 3.

## Data Availability

The datasets used and analysed in this study are available from the corresponding author on reasonable request.

## Abbreviations

CFA: Confirmatory factor analysis
GIS: Geographic Information System
ICC: Intraclass correlation coefficient
NEWS-Y: Neighborhood Environment Walkability Scale for Youth
NEWS-Y-G: Neighbourhood Environment Walkability Scale for Youth-German
PA: Physical activity
SES: Socioeconomic status
WHO: World Health Organization
