# Supplementary material for: Adaptation and validation of the neighbourhood environment walkability scale for German-speaking youth (NEWS-Y-G)
Source: BMC Public Health. 2026 Feb 13;26:709. doi: 10.1186/s12889-026-26590-3 (PMC12931025; doi:10.1186/s12889-026-26590-3)
Supplement: Supplementary file 1 — Supplementary Material 1. [file 12889_2026_26590_MOESM1_ESM.pdf]

Neighbourhood Environment Walkability Scale – Youth – German (NEWS-Y-G)

Version für Jugendliche

Entwickelt im Rahmen des WALKI-MUC-Projekts

Kontakt

Daniel Scheller
Professur für Sport- und Gesundheitsdidaktik
Technische Universität München
Georg-Brauchle-Ring 60/62
80992 München
E-Mail: daniel.scheller@tum.de

Einleitung

Wir möchten gerne mehr über deine Nachbarschaft erfahren. Dazu haben wir ein paar Fragen für dich. Wir möchten gerne wissen, was dir an deiner Wohnumgebung gefällt und was vielleicht nicht so toll ist. Dazu brauchen wir deine Hilfe.

Uns ist es wichtig, dass du den Fragebogen ganz frei und ehrlich ausfüllen kannst. Deswegen bitten wir dich, deinen Namen NICHT auf den Fragebogen zu schreiben.

**1. Wie ist deine Postleitzahl?**<br>*Die Postleitzahl ist die Nummer, die man braucht, um einen Brief oder ein Paket an deine Wohnadresse zu schicken.*<br>*Die Postleitzahl besteht aus fünf Zahlen und man schreibt sie in Deutschland vor den Namen der Stadt oder des Ortes.*<br>*Jede Postleitzahl gehört zu einem bestimmten Gebiet. In München beginnt die Postleitzahl mit 8.... zum Beispiel 80331.*	__ __ __ __ __<br>Deine Postleitzahl
**2. Falls du deine Postleitzahl nicht kennst:**<br>**In welchem Stadtviertel wohnst du?**	___________________
**3. Seit wann wohnst du dort?**	◯ Seit meiner Geburt<br>◯ Seit ungefähr _____ Jahren
**4. Wie alt bist du?**	___________________
**5. Welches Geschlecht hast du?**	◯ männlich<br>◯ weiblich<br>◯ divers
**6. Wie groß bist du (in cm)?**	___________________
**7. Wie viel wiegst du (in kg)?**	___________________

8. Welchen Beruf hat dein Vater?
Wenn dein Vater aktuell nicht arbeitet oder in Rente ist, dann nenne bitte den Beruf, den er als letztes hatte.

____
Sein Beruf

Was macht dein Vater in diesem Beruf? Bitte beschreibe es kurz.

____
____
Beschreibung davon, was er dort macht

9. Welchen Beruf hat deine Mutter?
Wenn deine Mutter aktuell nicht arbeitet oder in Rente ist, dann nenne bitte den Beruf, den sie als letztes hatte.

____
Ihr Beruf

Was macht deine Mutter in diesem Beruf? Bitte beschreibe es kurz.

____
____
Beschreibung davon, was sie dort macht

10. Auf welche Schule gehst du?

- ◯ Mittelschule
- ◯ Realschule
- ◯ Gymnasium
- ◯ Fachoberschule (FOS) / Berufsoberschule (BOS)
- ◯ Gesamtschule
- ◯ Berufsschule
- ◯ Förderschule
- ◯ Andere Schule (z.B. Waldorfschule)

11. An wie vielen der letzten sieben Tage hast du dich für mindestens eine Stunde körperlich angestrengt?

Hinweis: Damit ist alles gemeint, bei dem du dich viel bewegst und dein Herz schneller schlägt. Das kann z.B. der Sport im Verein sein, oder wenn du dich mittags mit Anderen draußen zum spielen triffst oder einfach das Fahrrad nimmst, um irgendwohin zu kommen.

- ◯ 0 Tage
- ◯ 1 Tag
- ◯ 2 Tage
- ◯ 3 Tage
- ◯ 4 Tage
- ◯ 5 Tage
- ◯ 6 Tage
- ◯ 7 Tage

A. Geschäfte und andere öffentliche Einrichtungen in deiner Wohnumgebung

Wie lange dauert es ungefähr <u>zu Fuß</u>, von deinem Zuhause zu den <u>nächstgelegenen</u> Geschäften oder unten genannten Einrichtungen zu gehen? Bitte kreise die Zeit ein, die du brauchen würdest, auch wenn du dort normalerweise nicht hingehst. Bitte kreise in jeder Zeile nur eine Antwortmöglichkeit an.

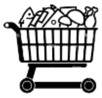

1. Kiosk / Kleiner Lebensmittelladen / Bäckerei	1-5 Min	6-10 Min	11-20 Min	21-30 Min	31+ Min	Weiß nicht
2. Supermarkt	1-5 Min	6-10 Min	11-20 Min	21-30 Min	31+ Min	Weiß nicht
3. Baumarkt	1-5 Min	6-10 Min	11-20 Min	21-30 Min	31+ Min	Weiß nicht
4. Obst- und Gemüseladen	1-5 Min	6-10 Min	11-20 Min	21-30 Min	31+ Min	Weiß nicht
5. Drogeriemarkt	1-5 Min	6-10 Min	11-20 Min	21-30 Min	31+ Min	Weiß nicht
6. Kleidungsgeschäft	1-5 Min	6-10 Min	11-20 Min	21-30 Min	31+ Min	Weiß nicht
7. Postfiliale	1-5 Min	6-10 Min	11-20 Min	21-30 Min	31+ Min	Weiß nicht
8. Bücherei	1-5 Min	6-10 Min	11-20 Min	21-30 Min	31+ Min	Weiß nicht
9. Kindergarten	1-5 Min	6-10 Min	11-20 Min	21-30 Min	31+ Min	Weiß nicht
10. Grundschule	1-5 Min	6-10 Min	11-20 Min	21-30 Min	31+ Min	Weiß nicht
11. Mittelschule / Realschule / Gymnasium	1-5 Min	6-10 Min	11-20 Min	21-30 Min	31+ Min	Weiß nicht
12. Buchhandlung	1-5 Min	6-10 Min	11-20 Min	21-30 Min	31+ Min	Weiß nicht
13. Fastfood-Restaurant / Schnellimbiss (z.B. Dönerladen) / Fastfood-Bude / Fastfood-Kette	1-5 Min	6-10 Min	11-20 Min	21-30 Min	31+ Min	Weiß nicht
14. Café / Eisdiele	1-5 Min	6-10 Min	11-20 Min	21-30 Min	31+ Min	Weiß nicht
15. Bank (z.B. Sparkasse)	1-5 Min	6-10 Min	11-20 Min	21-30 Min	31+ Min	Weiß nicht
16. Restaurant	1-5 Min	6-10 Min	11-20 Min	21-30 Min	31+ Min	Weiß nicht
17. Einkaufszentrum (viele Läden in einem Gebäude)	1-5 Min	6-10 Min	11-20 Min	21-30 Min	31+ Min	Weiß nicht
18. Apotheke	1-5 Min	6-10 Min	11-20 Min	21-30 Min	31+ Min	Weiß nicht
19. Friseur	1-5 Min	6-10 Min	11-20 Min	21-30 Min	31+ Min	Weiß nicht
20. Büros / Bürogebäude / Arbeitsstätten	1-5 Min	6-10 Min	11-20 Min	21-30 Min	31+ Min	Weiß nicht
21. Öffentliche Verkehrsmittel (z.B. Bus- / Tram- / U-Bahn- / S-Bahn- / Bahnstelle)	1-5 Min	6-10 Min	11-20 Min	21-30 Min	31+ Min	Weiß nicht

B. Freizeitanlagen in deiner Wohnumgebung

Wie lange dauert es ungefähr zu Fuß, von deinem Zuhause zu den nächstgelegenen unten genannten Freizeitanlagen zu gehen? Bitte kreise die Zeit ein, die du brauchen würdest, auch wenn du dort normalerweise nicht hingehst. Bitte kreise in jeder Zeile nur eine Antwortmöglichkeit an.

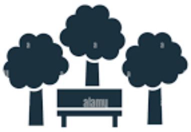

1. Überdachte Freizeitanlage / Sporthalle (öffentlich oder privat)	1-5 Min	6-10 Min	11-20 Min	21-30 Min	31+ Min	Weiß nicht
2. Fitnessstudio / Fitnesscenter / Gym (drinnen)	1-5 Min	6-10 Min	11-20 Min	21-30 Min	31+ Min	Weiß nicht
3. Calisthenics Park / Trainingspark / Fitnessparcour (draußen)	1-5 Min	6-10 Min	11-20 Min	21-30 Min	31+ Min	Weiß nicht
4. Strand / See / Fluss oder Bach	1-5 Min	6-10 Min	11-20 Min	21-30 Min	31+ Min	Weiß nicht
5. Wanderwege / Pfade / Wege für Fahrradtouren	1-5 Min	6-10 Min	11-20 Min	21-30 Min	31+ Min	Weiß nicht
6. Fußballplatz / Bolzplatz	1-5 Min	6-10 Min	11-20 Min	21-30 Min	31+ Min	Weiß nicht
7. Andere Sportplätze (z.B. Basketball- oder Skateplatz, Tischtennisplatten, Volleyballfeld oder Ähnliches)	1-5 Min	6-10 Min	11-20 Min	21-30 Min	31+ Min	Weiß nicht
8. Klettermöglichkeiten	1-5 Min	6-10 Min	11-20 Min	21-30 Min	31+ Min	Weiß nicht
9. Jugendzentrum / Jugendhaus / Jugendtreff	1-5 Min	6-10 Min	11-20 Min	21-30 Min	31+ Min	Weiß nicht
10. Schwimmbad / Freibad	1-5 Min	6-10 Min	11-20 Min	21-30 Min	31+ Min	Weiß nicht
11. Laufstrecke (z.B. Laufbahn)	1-5 Min	6-10 Min	11-20 Min	21-30 Min	31+ Min	Weiß nicht
12. Schule mit öffentlich zugänglichen Freizeiteinrichtungen (z.B. Pausenhof)	1-5 Min	6-10 Min	11-20 Min	21-30 Min	31+ Min	Weiß nicht
13. Öffentlicher Park	1-5 Min	6-10 Min	11-20 Min	21-30 Min	31+ Min	Weiß nicht
14. Öffentlicher Spielplatz mit Ausstattung	1-5 Min	6-10 Min	11-20 Min	21-30 Min	31+ Min	Weiß nicht
15. Öffentlicher Platz aus Gras / Sand / Erde, der kein Park ist (z.B. Wiese oder andere freie Flächen aus Beton oder ähnlichem Material)	1-5 Min	6-10 Min	11-20 Min	21-30 Min	31+ Min	Weiß nicht
16. Sitzbänke / Sitzgelegenheiten	1-5 Min	6-10 Min	11-20 Min	21-30 Min	31+ Min	Weiß nicht
17. Berg / Hügel	1-5 Min	6-10 Min	11-20 Min	21-30 Min	31+ Min	Weiß nicht

C. Arten von Wohngebäuden in deiner Wohnumgebung

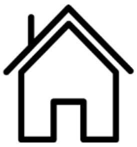

Bitte überlege dir, welche Art von Wohngebäuden es in deiner Wohnumgebung gibt und kreuze die Antwort an. Deine Wohnumgebung ist hier das Gebiet, das du von Zuhause fußläufig in 10-15 Minuten erreichen kannst. Bitte kreise in jeder Zeile nur eine Antwortmöglichkeit an.

1. Freistehende Einfamilienhäuser	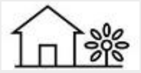	Keine	Wenige	Einige	Viele	Alle
2. Reihenhäuser	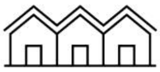	Keine	Wenige	Einige	Viele	Alle
3. Mehrfamilienhäuser oder Doppelhaushälften	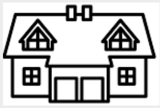	Keine	Wenige	Einige	Viele	Alle
4. Wohnblöcke oder Hochhäuser	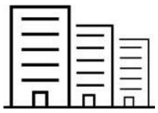	Keine	Wenige	Einige	Viele	Alle

D. Zugang zu Dienstleistungen

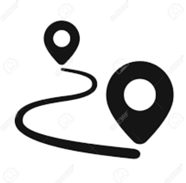

Bitte kreuze die Antwort an, die deine Wohnumgebung am besten beschreibt. Dabei bedeutet „zu Fuß erreichbar“, dass etwas 10-15 Minuten zu Fuß von deinem Zuhause entfernt ist. Bitte kreise in jeder Zeile nur eine Antwortmöglichkeit an.

1. Geschäfte sind leicht zu Fuß von meinem Zuhause zu erreichen.	Stimme gar nicht zu	Stimme eher nicht zu	Stimme eher zu	Stimme voll zu
2. Parken ist in den Einkaufsstraßen in meiner Wohnumgebung schwierig.	Stimme gar nicht zu	Stimme eher nicht zu	Stimme eher zu	Stimme voll zu
3. Ich kann von meinem Zuhause aus vieles zu Fuß erreichen (alleine oder mit jemandem).	Stimme gar nicht zu	Stimme eher nicht zu	Stimme eher zu	Stimme voll zu
4. Es ist einfach, von meinem Zuhause aus alleine oder mit jemandem zu einer Haltestelle der öffentlichen Verkehrsmittel (z.B. Bus-, Tram-, U-Bahn-, S-Bahn-, Bahnhaltestelle) zu gehen.	Stimme gar nicht zu	Stimme eher nicht zu	Stimme eher zu	Stimme voll zu
5. Die Straßen in meiner Wohnumgebung sind hügelig, was das Gehen erschwert.	Stimme gar nicht zu	Stimme eher nicht zu	Stimme eher zu	Stimme voll zu
6. In meiner Wohnumgebung gibt es viele Hindernisse, die es erschweren von A nach B zu kommen (z.B. Flüsse, Bahngleise, Autobahnen).	Stimme gar nicht zu	Stimme eher nicht zu	Stimme eher zu	Stimme voll zu

E. Straßen in meiner Wohnumgebung

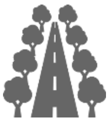

Bitte kreise die Antwort ein, die am besten auf deine Wohnumgebung (10-15 Minuten zu Fuß von deinem Zuhause) zutrifft. Bitte kreise in jeder Zeile nur eine Antwortmöglichkeit an.

1. Es gibt in meiner Wohnumgebung kaum Sackgassen.	Stimme gar nicht zu	Stimme eher nicht zu	Stimme eher zu	Stimme voll zu
2. Die Entfernung zwischen Kreuzungen in meiner Wohnumgebung ist meistens kurz (100 Meter oder weniger; die Länge eines Fußballfeldes oder weniger).	Stimme gar nicht zu	Stimme eher nicht zu	Stimme eher zu	Stimme voll zu
3. Es gibt viele verschiedene Wege, um in meiner Wohnumgebung von A nach B zu kommen. Ich muss nicht jedes Mal denselben Weg gehen.	Stimme gar nicht zu	Stimme eher nicht zu	Stimme eher zu	Stimme voll zu

F. Gehwege

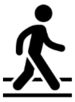

Bitte kreise die Antwort ein, die am besten auf deine Wohnumgebung (10-15 Minuten zu Fuß von deinem Zuhause) zutrifft. Bitte kreise in jeder Zeile nur eine Antwortmöglichkeit an.

1. Die meisten Straßen in meiner Wohnumgebung haben Gehwege.	Stimme gar nicht zu	Stimme eher nicht zu	Stimme eher zu	Stimme voll zu
2. Die Gehwege in meiner Wohnumgebung sind durch parkende Autos von der Straße / dem Verkehr getrennt.	Stimme gar nicht zu	Stimme eher nicht zu	Stimme eher zu	Stimme voll zu
3. Die Gehwege in meiner Wohnumgebung sind durch Gras / Erde / Grünstreifen von der Straße getrennt.	Stimme gar nicht zu	Stimme eher nicht zu	Stimme eher zu	Stimme voll zu
4. In meiner Wohnumgebung gibt es meistens Fahrradwege, die klar von den Gehwegen getrennt sind.	Stimme gar nicht zu	Stimme eher nicht zu	Stimme eher zu	Stimme voll zu

G. Zur Wohnumgebung

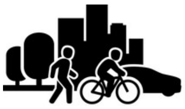

Bitte kreise die Antwort ein, die am besten auf deine Wohnumgebung (10-15 Minuten zu Fuß von deinem Zuhause) zutrifft. Bitte kreise in jeder Zeile nur eine Antwortmöglichkeit an.

1. In meiner Wohnumgebung gibt es Bäume entlang der Straßen.	Stimme gar nicht zu	Stimme eher nicht zu	Stimme eher zu	Stimme voll zu
2. Man kann viele interessante Dinge ansehen, während man durch meine Nachbarschaft geht (z.B. Brunnen, Hof, Statue).	Stimme gar nicht zu	Stimme eher nicht zu	Stimme eher zu	Stimme voll zu
3. In meiner Wohnumgebung gibt es viel Natur, die schön anzusehen ist (z.B. Gärten, Blumen, Tiere).	Stimme gar nicht zu	Stimme eher nicht zu	Stimme eher zu	Stimme voll zu
4. In meiner Wohnumgebung gibt es viele Gebäude / Häuser, die schön anzusehen sind.	Stimme gar nicht zu	Stimme eher nicht zu	Stimme eher zu	Stimme voll zu
5. In meiner Wohnumgebung liegt viel Müll herum.	Stimme gar nicht zu	Stimme eher nicht zu	Stimme eher zu	Stimme voll zu

H. Sicherheit in der Wohnumgebung

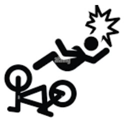

Bitte kreise die Antwort ein, die am besten auf deine Wohnumgebung (10-15 Minuten zu Fuß von deinem Zuhause) zutrifft. Bitte kreise in jeder Zeile nur eine Antwortmöglichkeit an.

1. In meiner Wohnumgebung gibt es auf den umliegenden Straßen so viel Verkehr, dass es schwierig oder unangenehm ist, dort (alleine oder mit jemandem) entlang zu gehen.	Stimme gar nicht zu	Stimme eher nicht zu	Stimme eher zu	Stimme voll zu
2. Die Verkehrsgeschwindigkeit auf den umliegenden Straßen ist meist gering (50km/h oder weniger).	Stimme gar nicht zu	Stimme eher nicht zu	Stimme eher zu	Stimme voll zu
3. Die meisten Fahrer überschreiten die ausgeschilderten Geschwindigkeitsbegrenzungen in meiner Wohnumgebung.	Stimme gar nicht zu	Stimme eher nicht zu	Stimme eher zu	Stimme voll zu
4. Die Straßen in meiner Wohnumgebung sind nachts gut beleuchtet.	Stimme gar nicht zu	Stimme eher nicht zu	Stimme eher zu	Stimme voll zu
5. Fußgänger und Fahrradfahrer können auf den Straßen in meiner Wohnumgebung leicht von Bewohnern in ihren Häusern gesehen werden.	Stimme gar nicht zu	Stimme eher nicht zu	Stimme eher zu	Stimme voll zu
6. In meiner Wohnumgebung gibt es auf viel befahrenen Straßen Zebrastreifen und Ampeln, die Fußgängern helfen, die Straße zu überqueren.	Stimme gar nicht zu	Stimme eher nicht zu	Stimme eher zu	Stimme voll zu
7. Wenn man in meiner Wohnumgebung zu Fuß unterwegs ist, merkt man, dass es viele Abgase gibt.	Stimme gar nicht zu	Stimme eher nicht zu	Stimme eher zu	Stimme voll zu
8. In meiner Wohnumgebung gibt es genügend schattige Plätze, um sich im Sommer abzukühlen.	Stimme gar nicht zu	Stimme eher nicht zu	Stimme eher zu	Stimme voll zu

I. Sicherheit vor Kriminalität

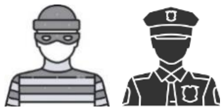

Bitte kreise die Antwort ein, die am besten auf dich und deine Wohnumgebung (10-15 Minuten zu Fuß von deinem Zuhause) zutrifft. Bitte kreise in jeder Zeile nur eine Antwortmöglichkeit an.

1. Wenn ich alleine draußen rund um mein Zuhause bin, fühle ich mich unwohl, weil ich vor manchen Menschen dort Angst habe.	Stimme gar nicht zu	Stimme eher nicht zu	Stimme eher zu	Stimme voll zu
2. Wenn ich mit einem Freund draußen rund um mein Zuhause bin, fühle ich mich unwohl, weil ich vor manchen Menschen dort Angst habe.	Stimme gar nicht zu	Stimme eher nicht zu	Stimme eher zu	Stimme voll zu
3. Wenn ich alleine oder mit Freunden draußen in meiner Wohnumgebung und den umliegenden Straßen unterwegs bin, fühle ich mich unwohl, weil ich vor manchen Menschen dort Angst habe.	Stimme gar nicht zu	Stimme eher nicht zu	Stimme eher zu	Stimme voll zu
4. Wenn ich in einem naheliegendem Park bin, fühle ich mich unwohl, weil ich vor manchen Menschen dort Angst habe.	Stimme gar nicht zu	Stimme eher nicht zu	Stimme eher zu	Stimme voll zu
5. In meiner Wohnumgebung werden immer wieder Straftaten begangen.	Stimme gar nicht zu	Stimme eher nicht zu	Stimme eher zu	Stimme voll zu
6. Aufgrund der Straftaten in meiner Wohnumgebung ist es nicht sicher, nachts alleine oder mit jemandem spazieren zu gehen.	Stimme gar nicht zu	Stimme eher nicht zu	Stimme eher zu	Stimme voll zu
