# Supplementary material for: Adaptation and validation of the neighbourhood environment walkability scale for German-speaking youth (NEWS-Y-G)
Source: BMC Public Health. 2026 Feb 13;26:709. doi: 10.1186/s12889-026-26590-3 (PMC12931025; doi:10.1186/s12889-026-26590-3)
Supplement: Supplementary file 2 — Supplementary Material 2. [file 12889_2026_26590_MOESM2_ESM.pdf]

# Neighbourhood Environment Walkability Scale – Youth – German

# (NEWS-Y-G)

## Cognitive Interview Guide

Developed as part of the WALKI-MUC project

Contact
Daniel Scheller
Associate Professorship of Didactics in Sport and Health
Technical University of Munich
Georg-Brauchle-Ring 60/62
80992 Munich
E-Mail: daniel.scheller@tum.de

Before completing the questionnaire:

Location: ________________________

Time and Date: ________________________

ID: ________________________

Given instructions to participants:

1. Mark any questions that you find difficult to answer while filling out the questionnaire
2. Underline any words you don't understand
3. Record the time: _________ minutes

After completing the questionnaire:

„Why did you find question X difficult to answer?“

*Once the question is understood:* "How could the question be phrased so that you would understand it immediately?"

________________________________________________________________________

________________________________________________________________________

________________________________________________________________________

________________________________________________________________________

________________________________________________________________________

________________________________________________________________________

________________________________________________________________________

________________________________________________________________________

________________________________________________________________________

________________________________________________________________________

________________________________________________________________________

________________________________________________________________________

________________________________________________________________________

Page 2 / Introduction Q11:
"How did you understand the question?" or "Why did you choose the scale value 4?" or "What activities did you think of when answering this question?"

______________________________________________________________________

______________________________________________________________________

Page 4 / B 15:
"What public place, other than a park, were you thinking of?"

______________________________________________________________________

______________________________________________________________________

B 17:
"What do you understand by a hill?" or "What came to mind when you thought of a hill?" *Ask for an example.*

______________________________________________________________________

______________________________________________________________________

Page 5 / Section C:
"How sure are you about the frequency of detached single-family homes in your neighbourhood?" *Ask about each housing type individually.*
Scale: 1 (very sure) to 5 (very unsure); "How did you approach answering this question?"
Detached single-family houses: ____
Terraced houses: ____
Apartment buildings or semi-detached houses: ____
Apartment complexes or highrises: ___

*If the scale value is (3, 4, or 5):* "Why are you not sure?"

______________________________________________________________________

______________________________________________________________________

D 2:
"Why did you choose this answer?" or "Do you know what the word SHOPPING AREAS means? Can you explain it?"

______________________________________________________________________

______________________________________________________________________

Page 6 / E 2:

"How sure are you in your answer?" Scale: 1 (very sure) to 5 (very unsure); "How did you approach answering this question?"

___

*If the scale value is (3, 4, or 5):* "Why are you not sure?"

___

___

G 3:

"What beautiful things in your neighbourhood were you thinking of?"

___

___

*If "Strongly Disagree" is ticked:* "What beautiful things did you think of?"

___

___

Page 7 / H 2:

"Do you know what 50 km/h or TRAFFIC SPEED means? Can you explain it?" ("How did you understand the question?" or "Why did you choose the scale value 4?"

___

H 3: "How do you notice that the posted speed limits are being exceeded?" "Do you know what the word SPEED LIMIT means? Can you explain it?" ("How did you understand the question?" or "Why did you choose the scale value 4?")

___

___

H 7: "How would you notice that there are a lot of exhaust fumes?"

___

___

H 8: "What places were you thinking of?" "Can you explain that in your own words?" ("How did you understand the question?" or "Why did you choose the scale value 4?")

I 5:
"Do you know what CRIMINAL OFFENCES means? Can you explain it?" ("How did you understand the question?" or "Why did you choose the scale value 4?")

"What people make you feel afraid?"

*If all answers are "Strongly Disagree":* "What people would make you feel afraid?"

*If answers differ for I1 to I3* (differences when alone or with someone):
"Why did you choose different answers?" "How did you understand the questions?" or "Why did you choose the scale value 4 for one question and 3 for the next?"

Other notes:
