# Supplementary material for: Adaptation and validation of the neighbourhood environment walkability scale for German-speaking youth (NEWS-Y-G)
Source: BMC Public Health. 2026 Feb 13;26:709. doi: 10.1186/s12889-026-26590-3 (PMC12931025; doi:10.1186/s12889-026-26590-3)
Supplement: Supplementary file 3 — Supplementary Material 3. [file 12889_2026_26590_MOESM3_ESM.pdf]

Neighbourhood Environment Walkability Scale – Youth – German (NEWS-Y-G)

Version for Youth

Developed as part of the WALKI-MUC project

Contact

Daniel Scheller
Associate Professorship of Didactics in Sport and Health
Technical University of Munich
Georg-Brauchle-Ring 60/62
80992 Munich
E-Mail: daniel.scheller@tum.de

Introduction

We would like to find out more about your neighbourhood. We have a few questions for you. We would like to know what you like about your neighbourhood and what might not be so good. We need your help to do this.

It is important to us that you can fill out the questionnaire freely and honestly. That is why we ask you to NOT write your name on the questionnaire.

**1. What is your postcode?**<br>*The postcode is the number you need to send a letter or parcel to your home address.*<br>*The postcode consists of five numbers and in Germany it is written before the name of the town or city.*<br>*Each postcode belongs to a specific area. In Munich, the postcode begins with 8.... for example 80331.*	— — — — —<br>Your Postcode
**2. If you don't know your postcode: Which neighbourhood do you live in?**	__________________
**3. How long have you been living there until now?**	◯ Since my birth<br>◯ For approximately _____ years
**4. How old are you?**	__________________
**5. What gender are you?**	◯ male<br>◯ female<br>◯ diverse
**6. How tall are you (in cm)?**	__________________
**7. How much do you weigh (in kg)?**	__________________

8. What is your father's job?
If your father is currently not working or is retired, please state the last job he had.

____
His Job

What does your father do in this job? Please describe it briefly.

____
____
Description of what he does there

9. What is your mother's job?
If your mother is currently not working or is retired, please state the last job she had.

____
Her Job

What does your mother do in this job? Please describe it briefly.

____
____
Description of what she does there

10. Which school do you go to?

- ◯ Mittelschule
- ◯ Realschule
- ◯ Gymnasium
- ◯ Fachoberschule (FOS) / Berufsoberschule (BOS)
- ◯ Gesamtschule
- ◯ Berufsschule
- ◯ Förderschule
- ◯ Other school (z.B. Waldorfschule)

11. On how many of the last seven days did you engage in physical activity for at least one hour?

Note: This means any activity where you move a lot and your heart beats faster. This could be sports at the club, for example, or when you meet up with other people to play outside at lunchtime or simply take the bike to get somewhere.

- ◯ 0 Days
- ◯ 1 Day
- ◯ 2 Days
- ◯ 3 Days
- ◯ 4 Days
- ◯ 5 Days
- ◯ 6 Days
- ◯ 7 Days

A. Stores, shops and other public facilities in your neighbourhood

Approximately how long does it take you to walk from your home to the nearest shops or facilities listed below? Please mark the time it would take you, even if you don't normally go there. Please mark only one answer option in each row.

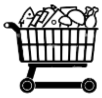

1. Kiosk, convenience store, bakery	1-5 Min	6-10 Min	11-20 Min	21-30 Min	31+ Min	Don't know
2. Supermarket	1-5 Min	6-10 Min	11-20 Min	21-30 Min	31+ Min	Don't know
3. Hardware store	1-5 Min	6-10 Min	11-20 Min	21-30 Min	31+ Min	Don't know
4. Fruit and vegetable store / market	1-5 Min	6-10 Min	11-20 Min	21-30 Min	31+ Min	Don't know
5. Drugstore	1-5 Min	6-10 Min	11-20 Min	21-30 Min	31+ Min	Don't know
6. Clothing store	1-5 Min	6-10 Min	11-20 Min	21-30 Min	31+ Min	Don't know
7. Post office	1-5 Min	6-10 Min	11-20 Min	21-30 Min	31+ Min	Don't know
8. Library	1-5 Min	6-10 Min	11-20 Min	21-30 Min	31+ Min	Don't know
9. Kindergarten	1-5 Min	6-10 Min	11-20 Min	21-30 Min	31+ Min	Don't know
10. Elementary school	1-5 Min	6-10 Min	11-20 Min	21-30 Min	31+ Min	Don't know
11. Middle school / high school	1-5 Min	6-10 Min	11-20 Min	21-30 Min	31+ Min	Don't know
12. Bookstore	1-5 Min	6-10 Min	11-20 Min	21-30 Min	31+ Min	Don't know
13. Fast food reastaurant / Snack bar / Food stall / Fast food chain	1-5 Min	6-10 Min	11-20 Min	21-30 Min	31+ Min	Don't know
14. Coffee shop / Ice-cream shop	1-5 Min	6-10 Min	11-20 Min	21-30 Min	31+ Min	Don't know
15. Bank (e.g. building society)	1-5 Min	6-10 Min	11-20 Min	21-30 Min	31+ Min	Don't know
16. Restaurant	1-5 Min	6-10 Min	11-20 Min	21-30 Min	31+ Min	Don't know
17. Shopping centre / Mall (many shops in one building)	1-5 Min	6-10 Min	11-20 Min	21-30 Min	31+ Min	Don't know
18. Pharmacy	1-5 Min	6-10 Min	11-20 Min	21-30 Min	31+ Min	Don't know
19. Hairdresser / barber shop	1-5 Min	6-10 Min	11-20 Min	21-30 Min	31+ Min	Don't know
20. Office buildings	1-5 Min	6-10 Min	11-20 Min	21-30 Min	31+ Min	Don't know
21. Public transport (e.g. Bus stop / Tram stop / Underground station / Train station)	1-5 Min	6-10 Min	11-20 Min	21-30 Min	31+ Min	Don't know

B. Recreational facilities in your neighbourhood

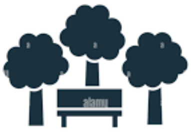

Approximately how long does it take you to walk from your home to the nearest leisure facilities listed below? Please mark the time it would take you, even if you don't normally go there. Please mark only one answer option in each row.

1. Indoor recreational facility / gym (public or private)	1-5 Min	6-10 Min	11-20 Min	21-30 Min	31+ Min	Don’t know
2. Fitness studio / fitness centre / gym (indoors)	1-5 Min	6-10 Min	11-20 Min	21-30 Min	31+ Min	Don’t know
3. Calisthenics park / training park / fitness trail (outdoor)	1-5 Min	6-10 Min	11-20 Min	21-30 Min	31+ Min	Don’t know
4. Beach / lake / river or creek	1-5 Min	6-10 Min	11-20 Min	21-30 Min	31+ Min	Don’t know
5. Bicycle paths / hiking paths / trails / paths	1-5 Min	6-10 Min	11-20 Min	21-30 Min	31+ Min	Don’t know
6. Soccer pitch / soccer field	1-5 Min	6-10 Min	11-20 Min	21-30 Min	31+ Min	Don’t know
7. Other sports fields (e.g. basketball or skateboard court, table tennis tables, volleyball court or similar)	1-5 Min	6-10 Min	11-20 Min	21-30 Min	31+ Min	Don’t know
8. Climbing facilities	1-5 Min	6-10 Min	11-20 Min	21-30 Min	31+ Min	Don’t know
9. Youth centre / youth house / youth club	1-5 Min	6-10 Min	11-20 Min	21-30 Min	31+ Min	Don’t know
10. Public swimming pool	1-5 Min	6-10 Min	11-20 Min	21-30 Min	31+ Min	Don’t know
11. Running track	1-5 Min	6-10 Min	11-20 Min	21-30 Min	31+ Min	Don’t know
12. School with publicly accessible recreational facilities (e.g. playground)	1-5 Min	6-10 Min	11-20 Min	21-30 Min	31+ Min	Don’t know
13. Public park	1-5 Min	6-10 Min	11-20 Min	21-30 Min	31+ Min	Don’t know
14. Public playground with equipment	1-5 Min	6-10 Min	11-20 Min	21-30 Min	31+ Min	Don’t know
15. Public space made of grass / sand / dirt that is not a park (e.g. meadow or other open space made of concrete or similar material)	1-5 Min	6-10 Min	11-20 Min	21-30 Min	31+ Min	Don’t know
16. Benches / seating	1-5 Min	6-10 Min	11-20 Min	21-30 Min	31+ Min	Don’t know
17. Mountain / hill	1-5 Min	6-10 Min	11-20 Min	21-30 Min	31+ Min	Don’t know

C. Types of residential buildings in your neighbourhood

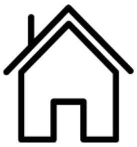

Please think about what kind of residential buildings there are in your neighbourhood and mark the answer. Your neighbourhood is the area that you can reach on foot from your home in 10-15 minutes. How common are the following types of homes in your neighbourhood? Please mark only one answer option in each row.

1. Detached single-family houses	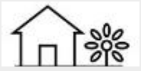	None	Few	Some	Many	All
2. Terraced houses	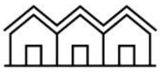	None	Few	Some	Many	All
3. Apartment buildings or semi-detached houses	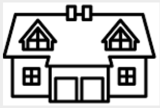	None	Few	Some	Many	All
4. Apartment complexes or highrises	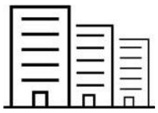	None	Few	Some	Many	All

D. Access to services

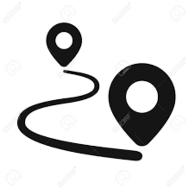

Please mark the answer that best describes your neighbourhood. "Within walking distance" means that something is a 10-15 minute walk from your home. Please mark only one answer option in each row.

1. Stores are within easy walking distance from my home.	Totally disagree	Rather disagree	Rather agree	Totally agree
2. Parking is difficult in shopping areas close to home.	Totally disagree	Rather disagree	Rather agree	Totally agree
3. I can reach many destinations from my home by foot (alone or with someone).	Totally disagree	Rather disagree	Rather agree	Totally agree
4. It is easy to reach public transportation (e.g., a bus, metro or train stop) by foot from my home, alone or with someone.	Totally disagree	Rather disagree	Rather agree	Totally agree
5. The streets in my neighbourhood are hilly, making it difficult to walk.	Totally disagree	Rather disagree	Rather agree	Totally agree
6. There are many obstacles in my neighbourhood making it difficult to get from place to place (e.g., rivers, train tracks, highways).	Totally disagree	Rather disagree	Rather agree	Totally agree

E. Streets in my neighbourhood

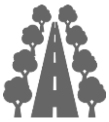

Please mark the answer that best applies to your neighbourhood (10-15 minutes walk from your home). Please mark only one answer option in each row.

1. There are very few cul-de-sac in my neighbourhood.	Totally disagree	Rather disagree	Rather agree	Totally agree
2. Distances between intersections in my neighbourhood are mostly short (100m or less, approximately the length of a soccer field).	Totally disagree	Rather disagree	Rather agree	Totally agree
3. There are many different ways to get from place to place in my neighbourhood. I don't have to take the same route every time.	Totally disagree	Rather disagree	Rather agree	Totally agree

F. Sidewalks

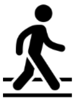

Please mark the answer that best applies to your neighbourhood (10-15 minutes walk from your home). Please mark only one answer option in each row.

1. Most streets in my neighbourhood have sidewalks.	Totally disagree	Rather disagree	Rather agree	Totally agree
2. The sidewalks in my neighbourhood are separated from the road / traffic by parked cars.	Totally disagree	Rather disagree	Rather agree	Totally agree
3. The sidewalks in my neighbourhood are separated from the road by grass / dirt / green strips.	Totally disagree	Rather disagree	Rather agree	Totally agree
4. In my neighbourhood, there are usually cycle paths that are clearly separated from the sidewalks.	Totally disagree	Rather disagree	Rather agree	Totally agree

G. About your neighbourhood

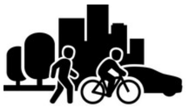

Please mark the answer that best applies to your neighbourhood (10-15 minutes walk from your home). Please mark only one answer option in each row.

1. There are trees along the streets in my neighbourhood.	Totally disagree	Rather disagree	Rather agree	Totally agree
2. You can look at many interesting things while walking through my neighbourhood.	Totally disagree	Rather disagree	Rather agree	Totally agree
3. There is a lot of nature in my neighbourhood that is beautiful to look at (e.g., gardens, flowers, animals).	Totally disagree	Rather disagree	Rather agree	Totally agree
4. There are many nice buildings / houses to look at in my neighbourhood.	Totally disagree	Rather disagree	Rather agree	Totally agree
5. There is a lot of litter lying around in my neighbourhood.	Totally disagree	Rather disagree	Rather agree	Totally agree

H. Safety in your neighbourhood

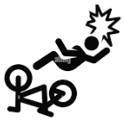

Please mark the answer that best applies to your neighbourhood (10-15 minutes walk from your home). Please mark only one answer option in each row.

1. In my neighbourhood, there is so much traffic on the surrounding streets that it is difficult or uncomfortable to walk down them (alone or with someone).	Totally disagree	Rather disagree	Rather agree	Totally agree
2. The traffic speed of surrounding streets is mostly low (less than 50km/h).	Totally disagree	Rather disagree	Rather agree	Totally agree
3. Most car drivers exceed the posted speed limit in my neighbourhood.	Totally disagree	Rather disagree	Rather agree	Totally agree
4. The streets in my neighbourhood are well lit at night.	Totally disagree	Rather disagree	Rather agree	Totally agree
5. Pedestrians and bicyclists can easily be seen on the streets in my neighbourhood by people in their homes.	Totally disagree	Rather disagree	Rather agree	Totally agree
6. There are crosswalks and traffic lights that help pedestrains cross busy streets in my neighbourhood.	Totally disagree	Rather disagree	Rather agree	Totally agree
7. While walking in my neighbourhood, you notice a lot of exhaust fumes.	Totally disagree	Rather disagree	Rather agree	Totally agree
8. In my neighbourhood there are enough of shady places to cool off in summer.	Totally disagree	Rather disagree	Rather agree	Totally agree

I. Safety against crime

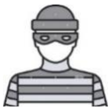

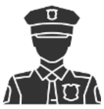

Please mark the answer that best applies to your neighbourhood (10-15 minutes walk from your home). Please mark only one answer option in each row.

1. When I'm alone outside around my home, I feel uncomfortable because I'm afraid of some of the people there.	Totally disagree	Rather disagree	Rather agree	Totally agree
2. When I'm outside with a friend around my home, I feel uncomfortable because I'm afraid of some of the people there.	Totally disagree	Rather disagree	Rather agree	Totally agree
3. When I'm out alone or with friends in my neighbourhood and the surrounding streets, I feel uncomfortable because I'm afraid of some of the people there.	Totally disagree	Rather disagree	Rather agree	Totally agree
4. When I'm in a nearby park, I feel uncomfortable because I'm afraid of some of the people there.	Totally disagree	Rather disagree	Rather agree	Totally agree
5. Criminal offences are repeatedly committed in my neighbourhood.	Totally disagree	Rather disagree	Rather agree	Totally agree
6. It is not safe to walk alone or with someone at night because of the criminal offences in my neighbourhood.	Totally disagree	Rather disagree	Rather agree	Totally agree
